# Stress‐linked morphological change associated with rearing techniques of hatchery‐reliant endemic landlocked Atlantic salmon ( *Salmo salar* m. *sebago* )

**DOI:** 10.1111/jfb.70149

**Published:** 2025-07-24

**Authors:** Aurora Hatanpää, Hannu Huuskonen, Jorma Piironen, Raine Kortet, William Bernard Perry

**Affiliations:** ^1^ Natural Resources Institute Finland (Luke), Natural Resources, Migratory Fish and Regulated Rivers Joensuu Finland; ^2^ University of Eastern Finland Department of Environmental and Biological Sciences Joensuu Finland; ^3^ Water Research Institute, School of Biosciences Cardiff University Cardiff UK; ^4^ Molecular Ecology and Evolution at Bangor, School of Biological Sciences Bangor University Bangor UK

**Keywords:** asymmetry, domestication, eye width, geometric morphometrics, hatchery, lower jaw, pectoral fin

## Abstract

Domestication effects caused by hatchery rearing impact various traits in fishes. Lake Saimaa landlocked salmon (*Salmo salar* m. *sebago*) have been dependent on hatchery propagation for over 50 years. The population is therefore at risk of hatchery‐induced phenotypes, which can be suboptimal in the wild, thus impacting the long‐term viability of the already critically endangered population. To assess the impact of rearing techniques, one‐summer‐old landlocked salmon morphology and asymmetry (an indicator of stress) was compared between four different hatchery rearing backgrounds, with all fish originating from the same family groups. The first group was maintained under standard hatchery conditions, the second under enriched rearing conditions (varying water level, direction and velocity), the third in semi‐natural hatchery conditions (outdoor stream ponds and natural prey) and the fourth group experienced wild conditions (stocked in the River Ala‐Koitajoki as alevins in spring and electrofished in September). Fish were photographed from both left and right sides and morphology was compared between rearing types using classical linear measurements as well as geometric morphometrics. Results indicate that natural conditions produce more symmetrical fish that have longer pectoral fins than their hatchery conspecifics, whereas fish reared in semi‐natural conditions show increased asymmetry. These results suggest that different rearing types elicit varying levels of stress, and that conservation efforts encouraging early‐stage stocking in the wild could be recommended when natural reproduction is not possible.

## 1 INTRODUCTION

Over the past century, human activities around the globe have threatened and brought to extinction numerous migratory salmonid populations by blocking access to their reproduction areas (Lundqvist et al., 2008). These actions have left many populations relying heavily on supportive stocking (Brown & Day, 2002; Fraser, 2008). Artificial propagation and hatchery rearing favour different traits than the natural environment and can lead to domesticated traits in short periods of time (Christie et al., 2012; Frankham, 2008; Hindar et al., 1991; Reisenbichler & Rubin, 1999; Waples, 1991). Soft domestication (i.e. not under direct active artificial selection) introduced by hatchery rearing of fishes causes these individuals to have a lower survival rate in the natural environment when compared to wild conspecifics (Araki et al., 2007, 2008; Brown et al., 2003; Norrgård et al., 2014; Wang & Ryman, 2001).

Reduction in the fitness of hatchery fish in the wild stems from hatchery practises that cause maladaptive changes in fish phenotypes, including morphological differences between hatchery‐reared and wild fish (Berejikian et al., 2000; Brockmark et al., 2010; Fleming et al., 1994; Law & Blake, 1996; Perry et al., 2019, 2020; Sánchez‐González & Nicieza, 2017). For example, body shape is important for swimming efficiency, foraging and predator avoidance (Jackson et al., 2011; Ojanguren & Braña, 2003; Pakkasmaa & Piironen, 2001), therefore changes in body morphology, caused by rearing in hatchery environments, can cause a loss of fitness in the wild. Physiological studies also indicate that fish in hatchery conditions can have poor swimming performance and weak cardiovascular health (Anttila et al., 2008; Frisk et al., 2020; Zhang et al., 2016). Fish are generally plastic in their early life, for example Stringwell et al. (2014) demonstrated that changes in hatchery fry morphology can happen as soon as 20 days after being released into the wild.

Phenotypic plasticity is common in salmonids (Hutchings, 1996; Morris et al., 2011) and can partly explain why populations with low genetic divergence display morphological differences (Garcia de Leaniz et al., 2007; Wang et al., 2002). Plasticity of salmonids can be seen in the morphological differences between rivers (Drian et al., 2012) along with local adaptation and ecological drivers (Rasmussen & Belk, 2017). In the same way, artificial rearing environments can induce changes in morphology. A healthy salmon population will have high levels of plasticity afforded both by a diverse gene pool as well as through individual phenotypic plasticity (Riddell & Swain, 1991). Plasticity is one of the factors that enables individuals and populations to cope with environmental changes without undergoing genetic change. If hatchery propagation is going to be an effective conservation tool for fish populations, especially in case of endangered populations, it should mimic the adaptations that would occur in the natural environment to secure increased fitness in the wild (Belk et al., 2008). Many elements of the hatchery environment, however, do not emulate wild conditions, including simplified environments and nutrition, as well as high stocking densities. These elements can cause stress, which can manifest itself in fluctuating morphological asymmetry, referred to as asymmetry from hereon. This type of asymmetry is characterised by small deviations from perfect bilateral symmetry in traits which would normally be symmetrical and has been suggested as a valuable biological indicator of environmental stress (Coda et al., 2017), especially in fish (Allenbach, 2011). Not only is this a useful indicator for studying stress in natural systems, but it also has more applied uses, such as assessing stress in the context of animal welfare (Knierim et al., 2007), therefore lower levels of stress in the rearing environment are predicted to result in individuals that are more symmetrical. Stress has been linked to lower reproductive fitness (Schreck & Tort, 2016), which can be an important issue that hinders reintroducing efforts.

Enriched rearing has been suggested as a partial solution to combat domestication caused by hatchery rearing and has been seen to increase fitness in Atlantic salmon (e.g. Hyvärinen & Rodewald, 2013; Karvonen et al., 2016; Rodewald et al., 2011). Enriched rearing involves fish being exposed to fluctuations in elements of their rearing environment from the egg stage to juveniles and even in adulthood. These fluctuations can include differences in water level, changes in the flow direction, changes in the feeding regimes and providing shelters to mimic the natural environment. Fish can also be reared in a semi‐natural environment where they have minimal human interaction and feed on natural prey (e.g. Hatanpää et al., 2020). However, this method is challenging as ensuring the proper level of nutrition is difficult and fish can also be subject to predation.

Here, we investigated the impact of rearing conditions on Lake Saimaa landlocked salmon (*Salmo salar* m. *sebago*) one‐summer‐old parr. Landlocked salmon possess adaptations, particularly relating to smoltification (Cairnduff et al., 2024), which allow them to survive despite being unable to undertake the key salmon life‐history trait, anadromy. Understanding the impact of hatchery‐rearing techniques on Lake Saimaa landlocked salmon is particularly important due to this population being entirely supported by stocking after natural stocks were destroyed during logging and the construction of hydropower stations in the 1950s and 1970s. At its smallest, the broodstock used to establish the hatchery stocks was below 10 individuals for four consecutive years in the early 1990s, but recent data indicate that this population still possesses genetic variation and adaptive potential for response to change from hatchery rearing back to more natural conditions (Janhunen et al., 2024). Ensuring hatchery‐rearing practices that minimise artificially induced morphological change, as well as stress, is a key to securing the longevity of this endemic population, which has been in existence since the last Ice Age (ca. 8000–10,000 years ago) (Piironen et al., 2013). We hypothesize that standard hatchery conditions will cause stress to individuals and thus morphological asymmetry, while enriching the hatchery environment will reduce levels of stress. To test these hypotheses, we compared four different rearing backgrounds: (i) standard hatchery rearing, (ii) enriched hatchery rearing, (iii) hatchery rearing in semi‐natural conditions in stream‐ponds and (iv) wild rearing in a natural stream.

## 2 MATERIALS AND METHODS

### 2.1 Ethics statement

Experimental procedures followed Animal Behavior Society (ABS) and the Association for the Study of Animal Behaviour (ASAB) guidelines for ethical treatment of animals and comply with current Finnish legislation. The experiments were conducted under licence from the Finnish Animal Experiment Board (ESAVI/5361/04.10.07/2013).

### 2.2 The study fish and creation of experimental groups

**FIGURE 1:**
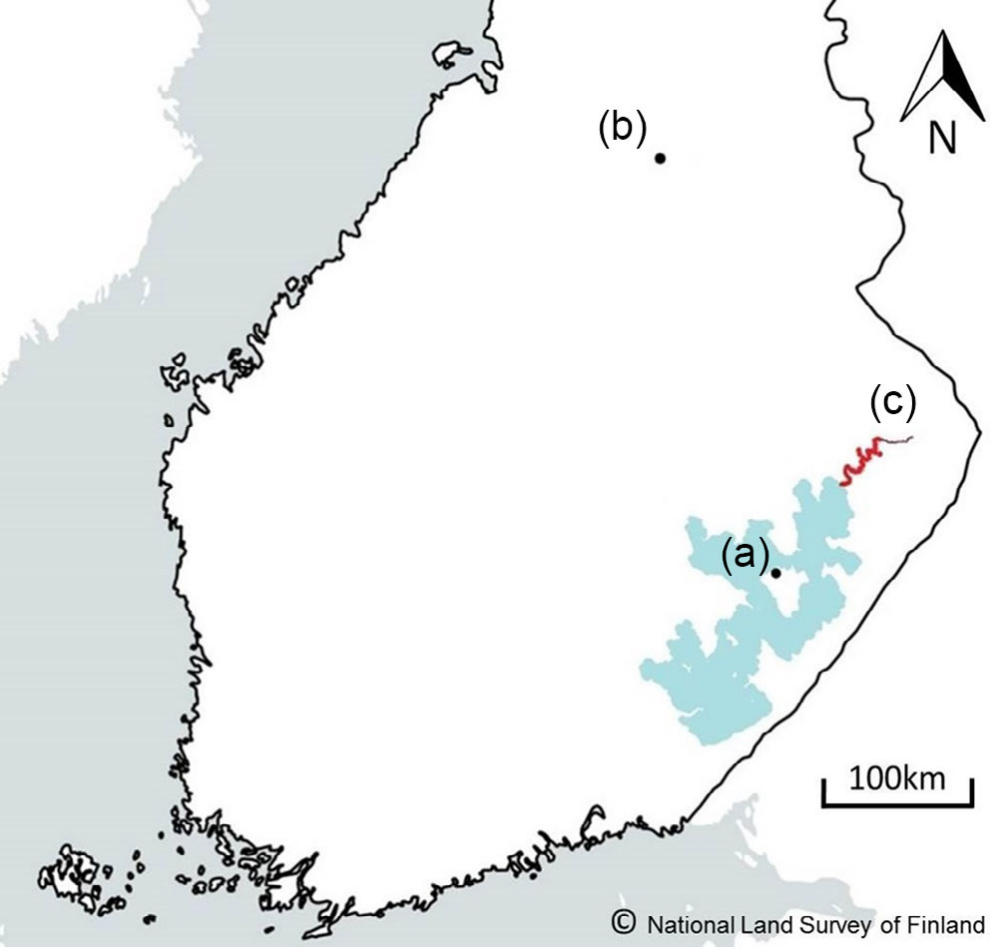
Map of southern Finland including locations relevant to the salmon sampled in this study: (a) the Enonkoski Aquaculture Station of the Natural Resources Institute, where fertilisation and early incubation of the fish occurred, (b) the Kainuu Fisheries Research Station of the Natural Resources Institute, where salmon were reared under standard, enriched and semi‐natural conditions post eyed‐egg stage, and (c) the River Ala‐Koitajoki, in red, where newly hatched alevins were released and later caught by electrofishing, making up the wild‐reared salmon. Morphological comparisons were then made between fish reared under these different conditions.

**FIGURE 2:**
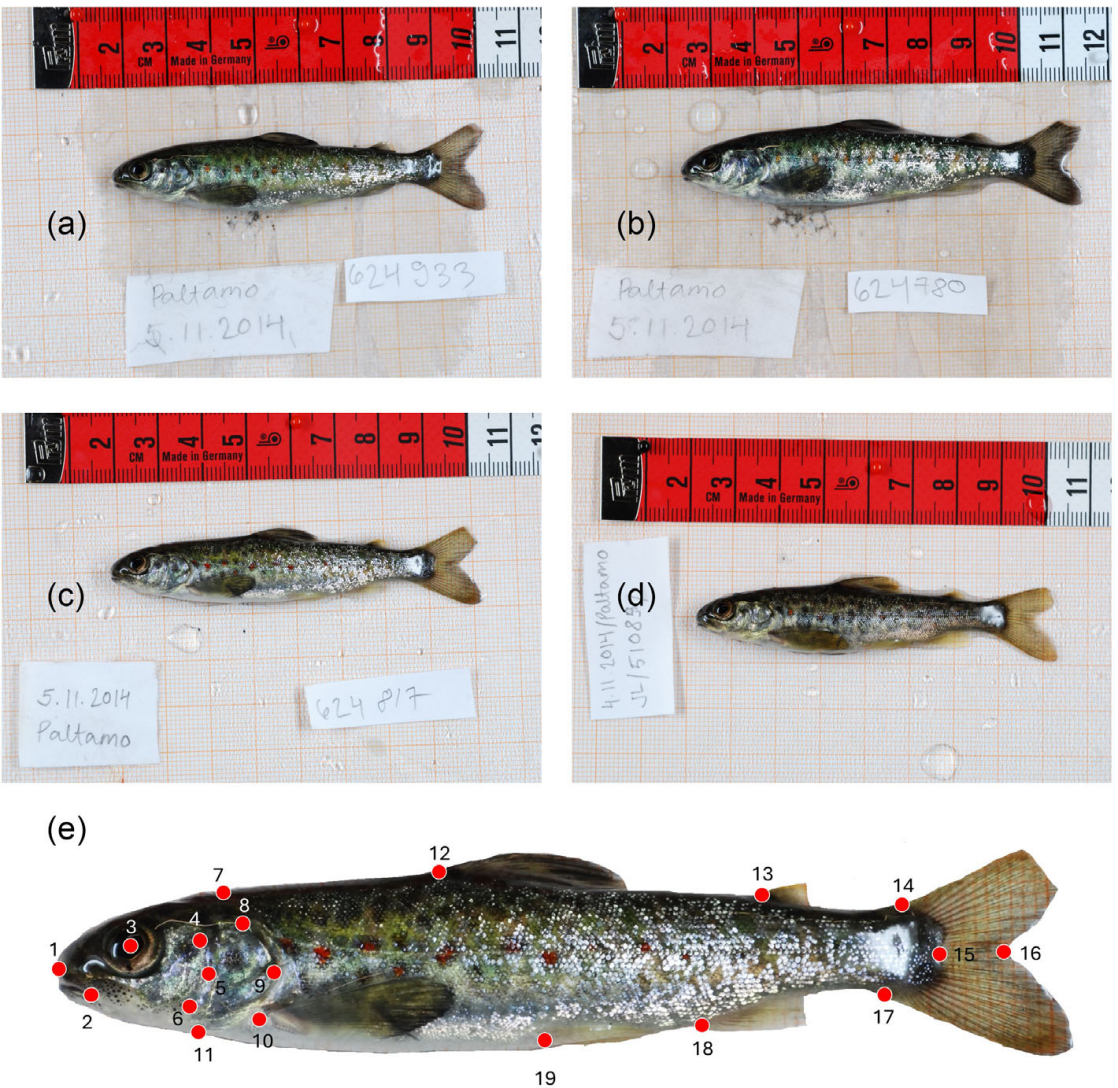
Photographs of Lake Saimaa landlocked salmon (*Salmo salar* m. *sebago*) used in the geometric morphometric and linear analyses in this study. These salmon include (a) standard‐reared, (b) enriched‐reared, (c) semi‐natural reared and (d) wild‐reared. Scales (in cm) are provided at the top of each photograph. The landmarks applied to these photographs used for the geometric morphometrics are shown in (e): landmark 1, anterior lip of the upper jaw; 2, intercept between the lower jaw and the maxilla; 3, centre of the eye; 4, dorsal limit of the preoperculum; 5, posterior limit of the preoperculum; 6, ventral limit of the preoperculum; 7, dorsal limit of the head; 8, dorsal limit of the operculum; 9, dorsal limit of the operculum; 10, insertion of the pectoral fin; 11, ventral limit of the head; 12, anterior dorsal fin insertion; 13, anterior insertion of adipose fin; 14, dorsal terminus of caudal flexure; 15, most anterior point of caudal peduncle; 16, most anterior point of caudal fin; 17, ventral terminus of the caudal flexure; 18, anterior insertion of anal fin; 19, anterior insertion of pelvic fin.

Eggs from a cultivated Lake Saimaa landlocked salmon broodstock were artificially fertilised in October 2013 (F1 hatchery generation) using 75 females from three year classes (2007, 2008 and 2009) and 75 males from four year classes (2008, 2009, 2010 and 2011). Five females and five males produced one fertilisation matrix (15 matrices in total, generating 375 families) to minimise possible inbreeding. This pooling of families made it possible to compare the rearing environment rather than genetic background. Fertilisation and early incubation occurred at the Enonkoski Aquaculture Station of the Natural Resources Institute, Finland (Figure 1a). At the eyed‐egg stage, the eggs pooled from all matrices were divided into four different treatments: (i) indoors standard‐reared (*N* = 7500, density 780 fish m^−2^, flow rate 0.2–0.5 L s^−1^, 3.2 m^2^ tanks, three replicates; Figure 2a), (ii) indoors enriched‐reared (*N* = 7500, density 780 fish m^−2^, flow rate 0.2–0.5 L s^−1^, 3.2 m^2^ tanks three replicates; Figure 2b), (iii) outdoors semi‐naturally reared fish (*N* = 7500, density 44 fish m^−2^, flow rate 3–10 L s^−1^, 39 m^2^ tanks, four replicates; Figure 2c), natural gravel bottom with natural zoobenthos) and (iv) juveniles to be stocked to natural stream as alevins (i.e. wild reared: *N* = 7500 eggs, density 0.2 fish m^−2^; Figure 2d). The eggs were marked with alizarin to later identify from wild fish. The wild‐reared fish were brought to the River Ala‐Koitajoki (6973832N, 673569E; Figure 1c) as newly hatched alevins on 2 May 2014 (water temperature + 3°C) and were left to grow under natural conditions until they were captured (64 individuals) by electrofishing (Bohlin et al., 1989) on 12 September 2014 (water temperature + 13°C). The River Ala‐Koitajoki is about 20 km long with 49‐m drop and with flow rate of 6 m^3^s^−1^ in summer. The fish fed on natural organisms occurring in the river habitat. After capture, the fish were transported to the Kainuu Fisheries Research Station (KFRS, www.kfrs.fi, Natural Resources Institute Finland; Figure 1b). The eyed eggs of the standard‐reared, enriched‐reared and semi‐natural fish were transported directly to KFRS. The fish were maintained under indoors standard, indoors enriched or outdoors semi‐natural conditions over the summer in 3.2 m^2^ glassfiber tanks where they were fed commercial feed with automatic feeders. Water temperature (2–17°C) followed outdoor conditions. Enriched fish were offered shelter from eyed‐egg stage onwards: first gravel and after starting to feed and swim independently they were offered submerged shelters. Enriched fish also experienced changes in the flow direction and water level in the tanks (Hyvärinen & Rodewald, 2013). Fish in semi‐natural conditions in concrete tanks with natural gravel bed foraged on natural food items (zoobenthos and drifting organisms) but had high mortality, partially due to occasional avian predation by white‐throated dippers (*Cinclus cinclus*), with only 119 individuals surviving. Standard‐ and enriched‐reared fish did not have significant mortality in the rearing tanks.

Between 12 and 16 September 2014, the fish were anaesthetised with benzocaine (40 mg L^−1^), measured (total length and wet mass) and tagged with individual PIT‐tags (12 mm HDX, Oregon RFID). After tagging, 30 haphazardly selected salmon (6–10 cm total length) from each rearing background were placed into eight 0.4 m^2^ circular holding tanks. The fish participated in feeding and swimming endurance experiments (see Hatanpää et al., 2020, the semi‐natural fish were excluded from Hatanpää et al. (2020) but they experienced the same treatment alongside rest of the study fish), after which (4–6 November 2024) they were euthanised with an overdose of benzocaine (200 mg l^−1^). Each fish was photographed (left and right lateral view) with a Nikon® D80 digital camera with a Nikon® AF Zoom Nikkor 28–85 mm f/3.5–4.5N lens. The photographs were taken from 25 cm distance using a fixed stand and constant camera settings (ISO‐800, focal length 60 mm, exposure time 1/60 sec, artificial fixed lighting, no flash). The tpsUtil software (Rohlf, 2010) was used to convert photographs into TPS files as well as to remove the effect of fish bending on the geometric morphometric analysis (Figure S1). To remove the effect of bending, the ‘unbend specimens’ function was used along with three landmarks along the lateral line of the fish (which were later removed) in addition to a landmark at the base of the tail (kept in the later analysis). Using tpsDig, 19 fixed landmarks were applied to photographs (by the same person, to avoid operator bias [Moccetti et al., 2023]) for geometric morphometrics to describe fish body shape and symmetry, while landmarks 1–11 were used for head shape (Figure 2e). Landmarks were selected based on multiple salmonid morphometric studies (including Knudsen et al., 2014; Moccetti et al., 2024; Rodger et al., 2025; Smith et al., 2017).

### 2.3 Geometric morphometrics

All statistical analyses were carried out in R 4.0.2 (R Core Team, 2021). Linear measurements and data are available at https://github.com/WillPerryMEFGL/salmon_asymmetry.git. Landmark data, for both the body and just the head, were analysed using the R package geomorph version 3.3.2 (Adams et al., 2003; Baken et al., 2021; Collyer & Adams, 2018, 2021), starting with a generalised Procrustes analysis to limit the effect of scale, orientation and translation between specimens. Aligned Procrustes coordinates were then used in a principal component analysis (PCA) using the function gm.prcomp, with visualisation of shape change along principal components achieved using thin‐plate splines using the function shape.predictor. Using the procD.lm function, a Procrustes analysis of variance (ANOVA) with permutation procedures (iterations = 999) was used to assess the effect of factors on shape. Factors included body length, which side of the sagittal plane the photograph was taken, rearing type, as well as their interaction terms. Rearing tank was also included as a random factor, nested within rearing type. Wild‐reared fish were not reared in tanks, but to harmonise the experimental approaches, and to allow for the random factor across all rearing types, wild‐reared fish were randomly assigned, in silico, to two groups, or ‘tanks’. Finally, pairwise comparisons of morphological disparity were conducted using the function morphol.disparity. Comparisons were made between rearing types, in addition to within rearing type comparisons between which side of the fish the photograph was taken, using 999 iterations and length as a covariate.

### 2.4 Linear measurements

Linear measurements were calculated using the distance between landmarks and the scale present in each of the photographs. To assess asymmetry, linear measurements taken on the right and left were subtracted from each other, with any negative numbers made absolute. Differences between left and right were then used as a response variable in a linear mixed effect model using the R package lme4 (Bates et al., 2015). The models included the factors rearing type and body length as well as rearing tank as a random factor, nested within rearing type. As with the geometric morphometrics, wild‐reared fish were randomly assigned, in silico, to two groups, or ‘tanks’. A separate linear model was constructed for pectoral fin length, lower jaw length and eye width. An ANOVA was then used to assess the effect of rearing type and body length on the different types of lengths, followed by a post hoc pairwise comparison (with Tukey multiple comparisons adjustment) between rearing types using the R package ‘emmeans’ (Lenth R. 2023).

In addition to asymmetry, averaged left and right linear length measurements were also assessed between rearing type. Separate linear regressions for the response variables mean pectoral fin length, mean lower jaw length and mean eye width were created, all of which had body length as the predictor variable. The residuals from each of these regressions were then used as a body length adjusted measure of the respective features. To assess the difference in means between rearing types, a linear mixed‐effect model was created for the response variables mean pectoral fin length, body length adjusted pectoral fin length, mean lower jaw length, body length adjusted lower jaw length, mean eye width and body length adjusted eye width, with rearing type included as a factor and tank nested in rearing type as a random factor. This was followed by an ANOVA and pairwise comparisons, as described above.

## 3 RESULTS

### 3.1 Geometric morphometrics: asymmetry

**FIGURE 3:**
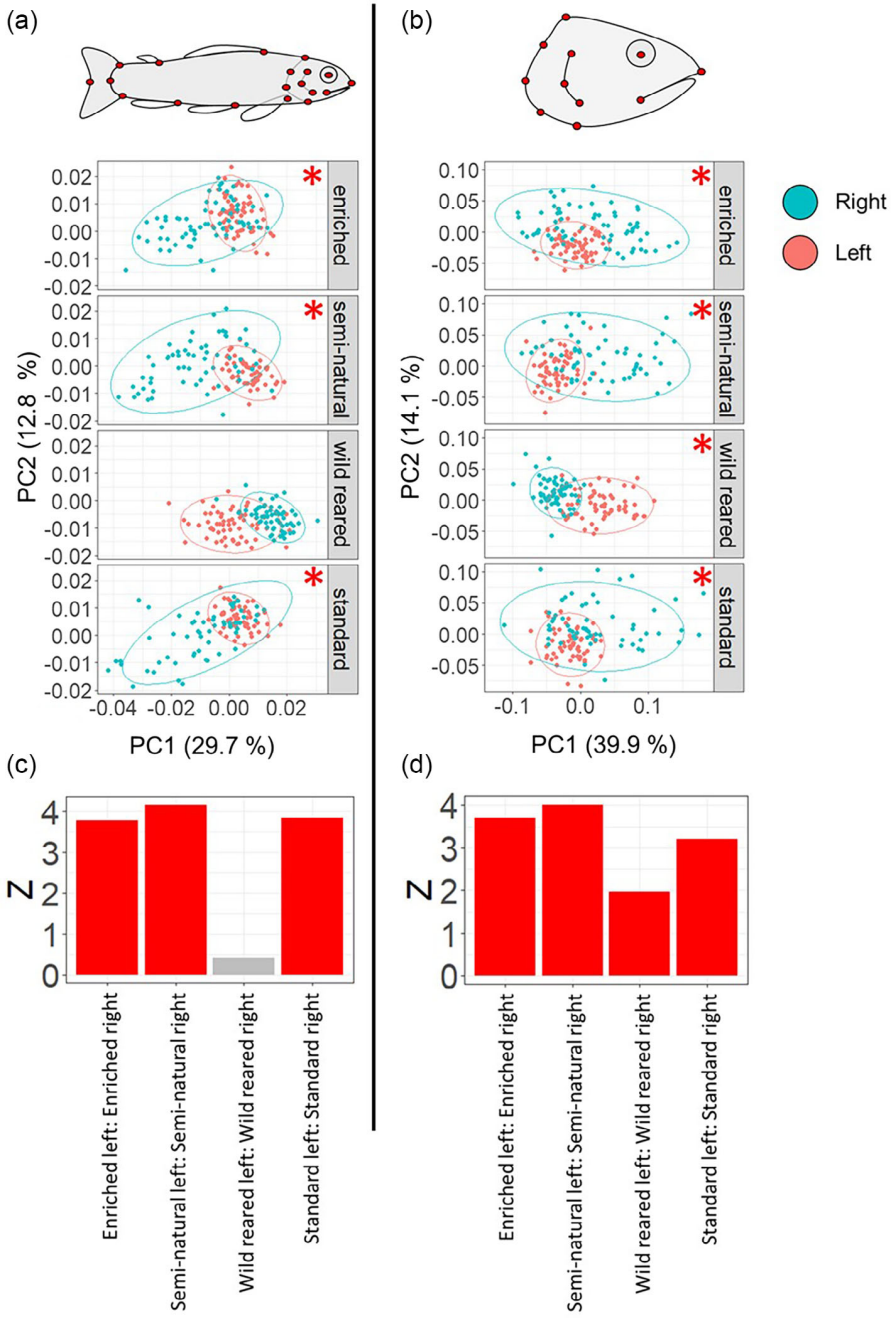
Outputs from the geometric morphometric analyses assessing (a, c) body and (b, d) head shape. Landmarks used in these analyses are shown as red dots on the diagrams of the salmon at the top of (a) and (b). Principal component analysis plots (a, b) summarise the shape from these landmarks, with each point representing an individual fish in. Points are split between rearing type and coloured by the side of the fish a photograph was taken on, right (blue) or left (red), along with 95% confidence ellipses around the points. Points closer together indicate a more similar shape. Also included are *z* scores from the pairwise comparisons, based on a Procrustes ANOVA, between sides, within rearing type, for (c) body and (d) head shape. Red asterisks and bars represent significant pairwise differences in the Procrustes ANOVA, which indicate that there is a difference in shape between the left and right side of the fish.

The side of the fish a photograph was taken on, rearing type and fish length all had a significant impact on body shape, with rearing type having the largest effect (Table 1 and Figure 3a). When looking at pairwise comparisons between the right‐ and left‐hand sides of the fish, there were significant differences in body morphology in the standard‐reared (*z* = 3.83, *p* < 0.01), enriched‐reared (*z* = 3.77, *p* < 0.01) and semi‐natural (*z* = 4.15, *p* < 0.01) groups, but not in the wild‐reared fish (*z* = 0.42, *p* = 0.36) (Figure 3c). The significant differences in enriched, semi‐natural and standard rearing types were largely driven by variation in the shape of the right side of the fish (Figure 3a). However, in the wild‐reared fish, where no significant difference was detected, there was much less variation in shape on the right side. Despite the lack of significant difference seen between sides in the wild‐reared fish, there is separation in the PCA. The reason this difference in shape between sides was not significant in the Procrustes ANOVA could be due to the influence of length in the model that the Procrustes ANOVA was based on, which would not have factored into the PCA.

**TABLE 1:** Output from Procrustes ANOVA analysis identifying the effect of factors on body and head shape.

Feature	Factor	*df*	Sum of squares	*R* ^2^	*Z*	*p*
Body	Side	1	0.013	0.054	6.58	0.001
	Rearing	3	0.028	0.111	9.88	0.001
	Length	1	0.005	0.019	5.42	0.001
	Side*rearing	3	0.026	0.106	7.58	0.001
	Side*length	1	0.001	0.004	2.98	0.004
	Rearing*length	3	0.002	0.008	2.82	0.002
	Side*rearing*length	3	0.001	0.005	0.89	0.183
Head	Side	1	0.139	0.046	5.97	0.001
	Rearing	3	0.135	0.045	5.90	0.001
	Length	1	0.025	0.008	2.89	0.003
	Side*rearing	3	0.328	0.108	7.78	0.001
	Side*length	1	0.010	0.003	1.57	0.064
	Rearing*length	3	0.017	0.006	0.74	0.23
	Side*rearing*length	3	0.017	0.006	0.75	0.22

The side of the fish a photograph was taken on, rearing type and fish length all had a significant impact on head shape, however, unlike body shape, the side the photo was taken on had the largest effect (Table 1 and Figure 3b). Pairwise comparisons showed that there were significant differences between the right‐ and left‐hand sides of the fish in all rearing types, including standard‐reared (*z* = 3.20, *p* < 0.01), enriched‐reared (*z* = 3.69, *p* < 0.01), semi‐natural (*z* = 4.02, p < 0.01) and wild‐reared (*z* = 1.96, *p* = 0.02). Unlike body shape, there was a significant difference between the left‐ and right‐hand sides of the wild‐reared fish, but the *z* score for this rearing type was considerably lower than for the other rearing types (Figure 3d).

### 3.2 Asymmetry in linear measurements: pectoral fin

**FIGURE 4:**
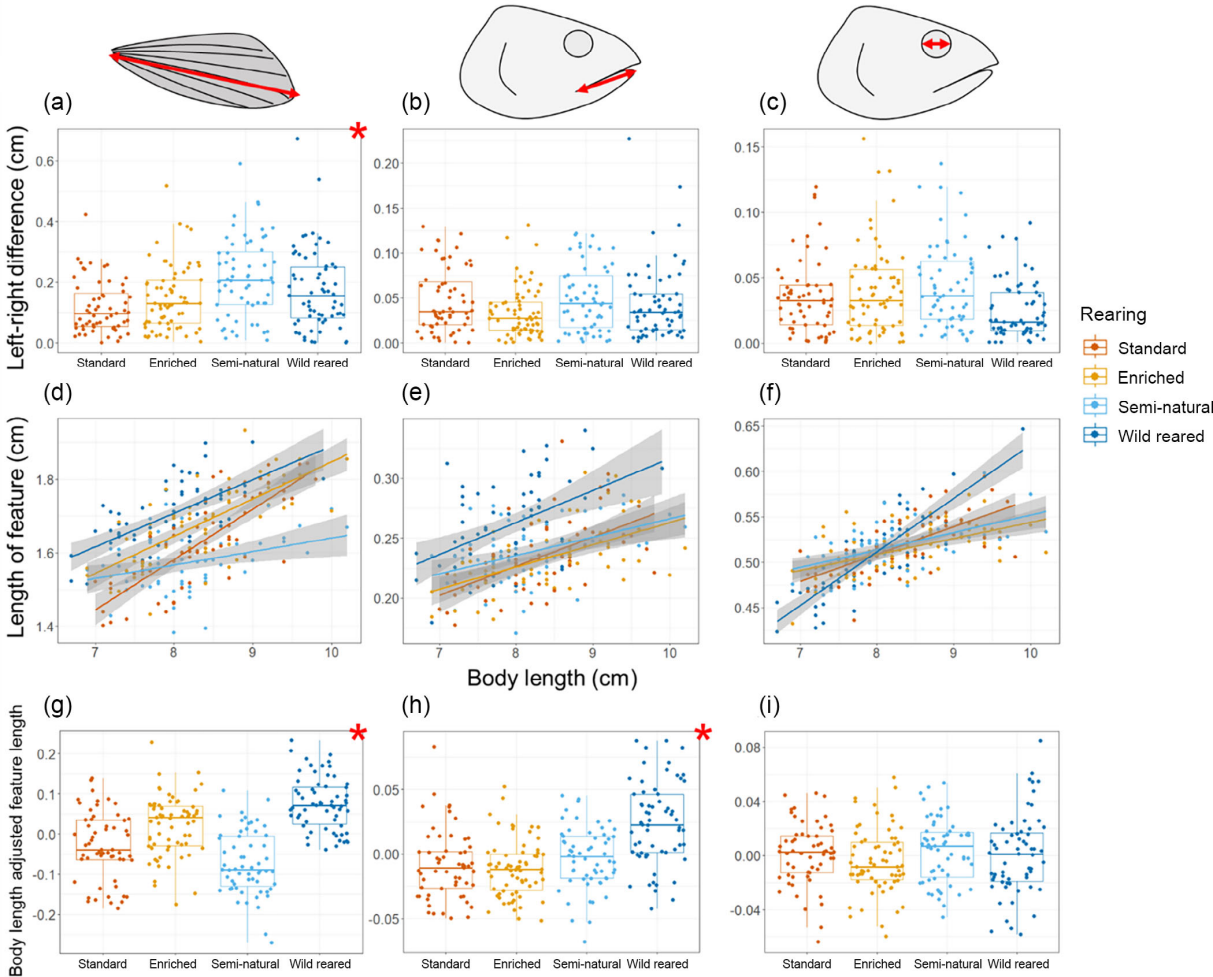
Boxplots and jitter plots for the differences in linear length between the left and right side of the fish coloured by rearing type for the (a) the pectoral fin, (b) the lower jaw length and (c) the eye width. The higher the value, the greater the difference between the left and right sides of an individual and therefore the greater asymmetry. In addition, regression plots show the relationship between mean (left and right) feature lengths and body length for (d) the pectoral fin, (e) the lower jaw and (f) the eye width, coloured by rearing type. All features showed a positive correlation with body length, with varying gradients and intercepts between rearing types. Finally, boxplots and jitter plots for body length adjusted mean feature lengths are provided for (g) the pectoral fin, (h) the lower jaw and (i) the eye width, coloured by rearing type. The higher the value, the larger the feature is relative to body size. Across all plots, each point represents an individual fish and they are coloured by rearing type. Red asterisks in panels containing boxplots represent a significant effect of rearing type, as tested with an ANOVA.

There was a significant effect of rearing on pectoral fin length difference between the left and right sides of the fish (*F*_3,9_ = 5.34, Sum Sq = 0.21, *p* = 0.02), and there was no significant effect of body length (*F*_1,236_ = 0.69, Sum Sq = 0.009, *p* = 0.41) (Figure 4a). The significant effect of rearing type was driven by a significant pairwise difference between the standard and semi‐natural reared types (*t*_24_ = 3.82, *p* < 0.01).

### 3.3 Linear measurements between rearing types: pectoral fin

There was a significant effect of rearing on pectoral fin length (*F*_3,235_ = 8.27, Sum Sq = 0.119, *p* < 0.01) and body length (*F*_1,240_ = 196.66, Sum Sq = 0.943, p < 0.01), with a significant interaction term (*F*_3,236_ = 9.59, Sum Sq = 0.138, *p* < 0.01) (Figure 4d). Rearing also had a significant effect on body length adjusted pectoral fin length (*F*_3,14_ = 17.80, Sum Sq = 0.28, *p* < 0.01), with significant pairwise differences between standard and semi‐natural (*t*_23_ = 2.82, *p* = 0.045), standard and wild (*t*_11_ = 4.33, *p* < 0.01), enriched and semi‐natural (*t*_22_ = 5.41, *p* < 0.01), and semi‐natural and wild (*t*_11_ = 6.51, *p* < 0.01) types (Figure 4g). Wild‐reared fish had the largest pectoral fin length relative to body length and semi‐natural reared fish had the smallest.

### 3.4 Asymmetry in linear measurements: lower jaw length

There was no significant effect of rearing (*F*_3,7_ = 2.59, Sum Sq = 0.010, *p* = 0.14) or body length (*F*_1,228_ = 3.45, Sum Sq = 0.004, *p* = 0.06) on lower jaw length difference between the left‐ and right‐hand sides of the fish (Figure 4b).

### 3.5 Linear measurements between rearing types: lower jaw length

There was no significant effect of rearing (*F*_3,240_ = 0.81, Sum Sq = 0.002, *p* = 0.49) on lower jaw length and there was a significant effect of body length (*F*_1,240_ = 78.99, Sum Sq = 0.054, *p* < 0.01), but no significant interaction term (*F*_3,240_ = 1.13, Sum Sq = 0.002, *p* = 0.34) (Figure 4e). However, rearing did have a significant effect on body length adjusted lower jaw length (*F*_3,244_ = 23.43, Sum Sq = 0.049, *p* < 0.01), with significant pairwise differences between standard and wild (*t*_6_ = 6.67, *p* < 0.01), enriched and wild (*t*_5_ = 7.41, *p* < 0.01), and semi‐natural and wild (*t*_5_ = 5.40, *p* = 0.01) types (Figure 4h). Wild‐reared fish had the largest lower jaw length relative to body length and enriched‐reared fish had the smallest.

### 3.6 Asymmetry in linear measurements: eye width

There was no overall significant effect of rearing (*F*_3,11_ = 1.75, Sum Sq = 0.004, *p* = 0.21) or body length (*F*_1,242_ = 0.04, Sum Sq = 0.00003, *p* = 0.84) on eye width difference between the left‐ and right‐hand sides of the fish (Figure 4c).

### 3.7 Linear measurements between rearing types: eye width

There was a significant effect of rearing (*F*_3,232_ = 17.43, Sum Sq = 0.026, *p* < 0.01) and body length (*F*_1,240_ = 225.84, Sum Sq = 0.111, *p* < 0.01) on eye width, with a significant interaction term (*F*_3,236_ = 18.07, Sum Sq = 0.027, *p* < 0.01) (Figure 4f). Rearing did not have a significant effect on body length adjusted eye width (*F*_3,12_ = 0.90, Sum Sq = 0.002, *p* = 0.47) (Figure 4i).

## 4 DISCUSSION

Rearing fish in artificial environments has been demonstrated to induce profound effects on their biology, including morphology (Taylor, 1986; Von Cramon‐Taubadel et al., 2005; Wessel et al., 2006). Understanding plasticity in morphological development in artificial environments is vital in the context of conservation hatcheries, where fish are potentially released into the wild with suboptimal phenotypes, thus impacting their lifetime fitness (Solberg et al., 2020; Von Cramon‐Taubadel et al., 2005). Looking at morphological asymmetry can provide insight into the levels of stress caused by different rearing techniques. Using these ideas, we explored whether rearing techniques could impact the morphology of the critically endangered endemic Lake Saimaa landlocked salmon, which are entirely reliant on hatchery rearing (Hatanpää et al., 2021). Our results indicate clear rearing‐dependent differences in body morphology, pectoral fin length and lower jaw length. Moreover, we demonstrate profound rearing‐dependent differences in asymmetry, suggesting that hatchery practices cause stress during the first year of salmon rearing.

### 4.1 Asymmetry

Asymmetry as a proxy for increased developmental instability, mediated by energy allocation in growing organisms, can be a direct consequence of stress and is connected with reduced fitness (e.g. Lajus et al., 2014, 2019). In the present data, significant left–right asymmetry in body shape was seen in all rearing types apart from those that were wild‐reared, with the PCA also showing lower variance in body shape in the wild‐reared group (Figure 3a,c). A similar trend was also seen for head shape (Figure 3b,d). The most symmetrical group was therefore the one that was reared in a natural river since their alevin stage. The most asymmetrical rearing type, when it came to the *z* scores, was semi‐natural. This observed variation in the morphology between rearing types was caused by the rearing environment rather than genetic background, as all the groups originated from the same pooled families.

Asymmetry in body and head shape seen in enriched, semi‐natural and standard rearing appears to be driven by the right side having far greater shape variation than the left side. In addition to stress, this result could be due to these fish being reared in circular tanks which generate circular flow that the fish swim against. Circular flow is created by water entering through a pipe positioned on the tank wall and then leaving through a central drain at the bottom of the tank. Such a design is widely adopted in aquaculture settings as it provides more stable flow patterns, prevents anoxic zones and helps with waste collection (Oca & Masalo, 2013). The design also creates a flow velocity gradient, with higher flow velocities on the outer edge of the tank and lower flow velocities at the centre of the tank (Sin et al., 2021). Therefore, because fish are swimming into the flow, they will be experiencing asymmetric flow velocities between their left and right sides, which could be influencing asymmetry in body and head shape. Direction of the circular flow (clockwise or anticlockwise) may not be important because enriched‐reared fish experienced mixed directional flows and still presented with a highly variable shape on the right side. What may be more important is the lack of complex disrupted flow such as eddies and riffles that would be found in the natural environment, as experienced by the wild‐reared fish. It is important to note that, while wild‐reared fish had the lowest z scores for differences in left–right head shape, there was still a significant difference between the two sides, unlike body shape. Although the incongruence between significant asymmetries in the body and head shape could have a biological cause, is it more likely to be methodological. The head occupies a far smaller space in photographs yet contains more landmarks than the rest of the body. In addition to limited image resolution of the head, some structures on the head are less finite than those located on the body and may be harder to place. These two factors mean that there will be greater noise in the head shape data, which may be contributing to the wild‐reared significant asymmetry result.

Like body and head shape, asymmetry in length measurements was most pronounced in fish reared under semi‐natural conditions, specifically the pectoral fins, suggesting that the semi‐natural rearing environment induced high levels of stress. When compared to the two other hatchery‐rearing treatments that occurred indoors (standard and enriched), the outdoor environment utilised in the semi‐natural rearing might have caused intensified stress in the early phase of the experiment when the fish were transferred, already in their eyed‐egg stage, to this new environment. These outdoor fish foraged on natural food items but were also exposed to occasional avian predation (e.g. white‐throated dippers *Cinclus cinclus*), with nutrition and predation being well‐known elicitors of stress (Hawkins et al., 2004). The present results add to the evidence that, to avoid possibly maladaptive asymmetrical phenotypic traits, hatcheries should opt for wild early‐rearing strategies over rearing in more artificial environments, even if those artificial environments have alterations to make them more natural. For example, although predation is a natural process that would occur in the wild, the stress it causes appears to be compounded when combined with more simple and homogeneous artificial rearing in an environment that has fewer hiding spots and chances of escape.

The lowest levels of asymmetry in linear measurements would be expected in the wild‐reared fish, given they are not exposed to hatchery stressors. Contrary to the predication, wild‐reared fish showed the second most asymmetrical pectoral fins and hatchery fish showed the least amount of pectoral fin asymmetry (Figure 4a). Previous work on salmonid wild‐hatchery comparisons, in the context of caudal fins, has shown that wild fish do not always exhibit the lowest levels of asymmetry, specifically against enriched hatchery‐rearing types (Cogliati et al., 2023), although Cogliati et al. (2023) observed that standard hatchery‐reared fish had the greatest level of asymmetry, unlike the results in this study. Overall, what this could demonstrate is that asymmetry in fins is driven by acute physical stress, damage and regrowth, rather than stress‐induced developmental change associated with whole body shape. Additionally, these results also suggest that some asymmetry in fins is natural.

### 4.2 General morphology

Rearing had a significant effect on mean pectoral fin length and mean lower jaw length, relative to body length, with wild‐reared fish having the largest pectoral fin and lower jaw lengths (Figure 4g,h). Larger pectoral fin lengths are likely to be advantageous in the natural environment because of the strong and variable flow found in the river, with larger pectoral fins providing fish with greater stability when navigating (Arnold et al., 1991). Indeed, the second next largest pectoral fins, relative to body length, were found in the enriched group, where circular flow in the tank was routinely alternated, thus producing some variability. Standard and semi‐natural rearing had no variability in flow and had the lowest pectoral fin sizes relative to body length. Previous studies assessing the effect of rearing on Atlantic salmon pectoral fins have also demonstrated the same trend that is shown here, with larger fins seen in wild‐reared fish (Pelis & McCormick, 2003), although not unanimously (Stringwell et al., 2014). Differences seen between studies are likely due to differing experimental salmon stocks and thus genetic background, as well as variability in flow regimes between lotic environments. Larger fins have also been associated with enhanced predator avoidance in crucian carp (*Carassius carassius*), with longer fins associated with higher survival rate in the wild (Hulthén et al., 2024).

Larger lower jaws, relative to body size, were observed in wild‐reared fish, which could be more advantageous in the river environment where food sources are going to be more variable in both size and composition (Snorrason et al., 1994; Wankowski, 1979). Further evidence suggesting that food source has an impact on jaw morphology comes from the standard‐ and enriched‐reared fish, which were fed on artificial feed and had the smallest jaw sizes relative to body size. Fish reared semi‐naturally had larger jaw sizes (albeit not significantly) than standard‐ and enriched‐reared fish, and they were fed on more natural prey items. The reason that the jaw lengths of fish reared semi‐naturally were not more similar to those of the wild‐reared fish could be due to a lower diversity of natural food source available in the outdoor concrete pools, as well as food sources being scarcer.

Despite significant asymmetry being observed in eye width between rearing types, rearing type did not have a significant impact on mean eye width. Experiments in Arctic charr (*Salvelinus alpinus*) have demonstrated that eye size can vary as a plastic response to prey items (Adams et al., 2003) and experiments in Atlantic salmon have shown reductions in eye size linked with artificial rearing (Perry et al., 2021). The lack of eye width change seen in this study could suggest that eye width is less plastic in Atlantic salmon than in other salmonids or that the environmental heterogeneity between rearing types is not enough to elicit a plastic response.

Adding to the linear measurements, the geometric morphometric results showed that rearing environment impacted the body shape of the one‐summer‐old landlocked Atlantic salmon. This has been well established already in the literature among many fish species, induced by environmental drivers such as habitat complexity, temperature and food supply (Marcil et al., 2006; Pulcini et al., 2014; Stringwell et al., 2014). The body shapes of the semi‐naturally reared fish were most similar to the wild‐reared fish (Figures S2 and S3), which has been demonstrated previously in juvenile Chinook salmon (*Oncorhynchus tshawytscha*) (Cogliati et al., 2023). Like Cogliati et al. (2023), the similarity in shape between wild and semi‐naturally reared fish was largely explained by them both having narrower bodies when compared to standard‐ and enriched‐reared fish. Despite the semi‐natural rearing producing the most similar shape to wild rearing, semi‐natural fish displayed asymmetry in the geometric morphometrics, had the highest levels of asymmetry in the linear measurements and significantly deviated from wild‐reared fish in mean pectoral fin length and lower jaw length. This demonstrates the importance of looking beyond overall body shape when assessing which rearing method produces the most natural fish because body shape may just be reflecting which fish have similar body condition.

### 4.3 Methodological implications

Not only do the results presented here have important ramifications for the rearing of hatchery fish and understanding the role of plasticity in artificial environments, but they also have important methodological implications for morphology‐based studies on fish. Most experimental studies examining the morphology of fish will take photographs from one side (Arechavala‐Lopez et al., 2012; Ikpeme et al., 2017; Perriman et al., 2022). However, these results demonstrate that choosing which side of the fish to photograph could have a profound influence on the outcome of a study. It is therefore recommended that measurements should be taken on both sides of fish, and a mean taken, to ensure that asymmetry is not a confounding factor in experiments where it is not the sole focus of study.

## 5 CONCLUSION

Our results indicate that one‐summer‐old landlocked Atlantic salmon grown in a river under natural conditions were more symmetrical and had notably longer fins and lower jaws than their hatchery conspecifics. The large influence of rearing seen on morphology in this study indicates that although this population has low genetic diversity, the cohort has plasticity left in the population. Based on our results, we recommend that supportive stocking to wild environments is done at the earliest stages of fish development to help reduce potential stress caused by artificial rearing environments. In doing so, there is the potential to reduce possible developmental abnormalities in morphology that are caused by stress and the hatchery environment.

## AUTHOR CONTRIBUTIONS

**Aurora Hatanpää:** conceptualization, data collection and curation, formal analysis, validation, visualization, writing – original draft, writing – review and editing, funding acquisition. **Hannu Huuskonen:** Supervision, methodology, validation, visualization, writing – review and editing. **Raine Kortet:** Supervision, writing – review and editing, funding acquisition. **Jorma Piironen:** Conceptualization, methodology, validation, supervision, writing – review and editing, funding acquisition. **William B. Perry:** Conceptualization, data curation, software, formal analysis, investigation, writing original draft, writing – review and editing.

## FUNDING INFORMATION

The study was financially supported by the Natural Resources Institute of Finland, Ministry of Agriculture and Forestry of Finland, the Raija and Ossi Tuuliainen Foundation, and the Finnish Cultural Foundation.

## Supporting information


**FIGURE S1.** Principal component analysis summarising body shape, based on 19 landmarks, of all fish used in this study (a) before and (b) after being adjusted for fish bending. Each point represents a fish, coloured by the side the photograph was taken on, either left (red) or right (blue). Adjustment for fish bending was done using the tpsUtil software. Thin‐plate splines describing the shape summarised by each of the two main principal components at their minimum and maximum are also provided on the *x* and *y* axes. As can be seen in the thin‐plate splines, extremes of the principal components in (a) are characterised by bending along the anteroposterior axis, but this effect is removed in (b) after the adjustment.

**FIGURE S2.** Principal component analysis plots from the geometric morphometric analyses assessing (a) body and (b) head shape. Plots are split by the side a photograph was taken and coloured by rearing type. The 95% confidence ellipses can be seen around the data points, coloured by rearing type. These plots look to identify differences in body and head shape caused by rearing alone, regardless of asymmetry. Points closer together indicate a more similar shape.

**FIGURE S3.** Principal component analysis summarising body shape, based on 19 landmarks, of all fish used in this study, but with points only taken from the left side of the fish. The left side was chosen because it displayed less variation in principle component one, as shown in Figure S2. Points are coloured by rearing type. Points closer together indicate a more similar shape. Thin‐plate splines are present along the *x* and *y* axes, describing the shape summarised by each of the two main principal components at their minimum and maximum. Finally, a salmon diagram with the position of the landmarks (red points) which formed the basis of the shape analysis is also included.
