# Supplementary figures and images for: Stress‐linked morphological change associated with rearing techniques of hatchery‐reliant endemic landlocked Atlantic salmon ( Salmo salar m. sebago )
Source: J Fish Biol. 2025 Jul 24;107(5):1577–88. doi: 10.1111/jfb.70149 (PMC12710846; doi:10.1111/jfb.70149)

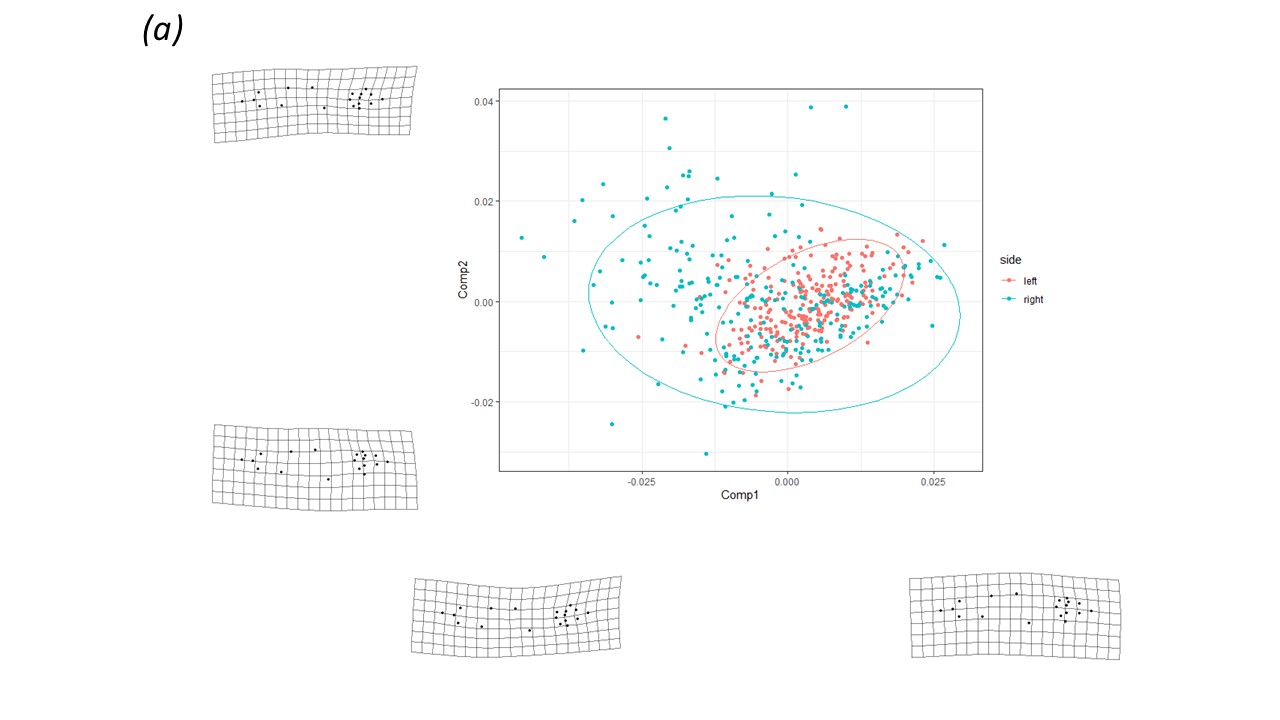

Supplement: Supplementary file 1 — FIGURE S1. Principal component analysis summarising body shape, based on 19 landmarks, of all fish used in this study (a) before and (b) after being adjusted for fish bending. Each point represents a fish, coloured by the side the photograph was taken on, either left (red) or right (blue). Adjustment for fish bending was done using the tpsUtil software. Thin‐plate splines describing the shape summarised by each of the two main principal components at their minimum and maximum are also provided on the x and y axes. As can be seen in the thin‐plate splines, extremes of the principal components in (a) are characterised by bending along the anteroposterior axis, but this effect is removed in (b) after the adjustment. [file JFB-107-1577-s002.zip › Supplementary Figure 1 A.jpg]

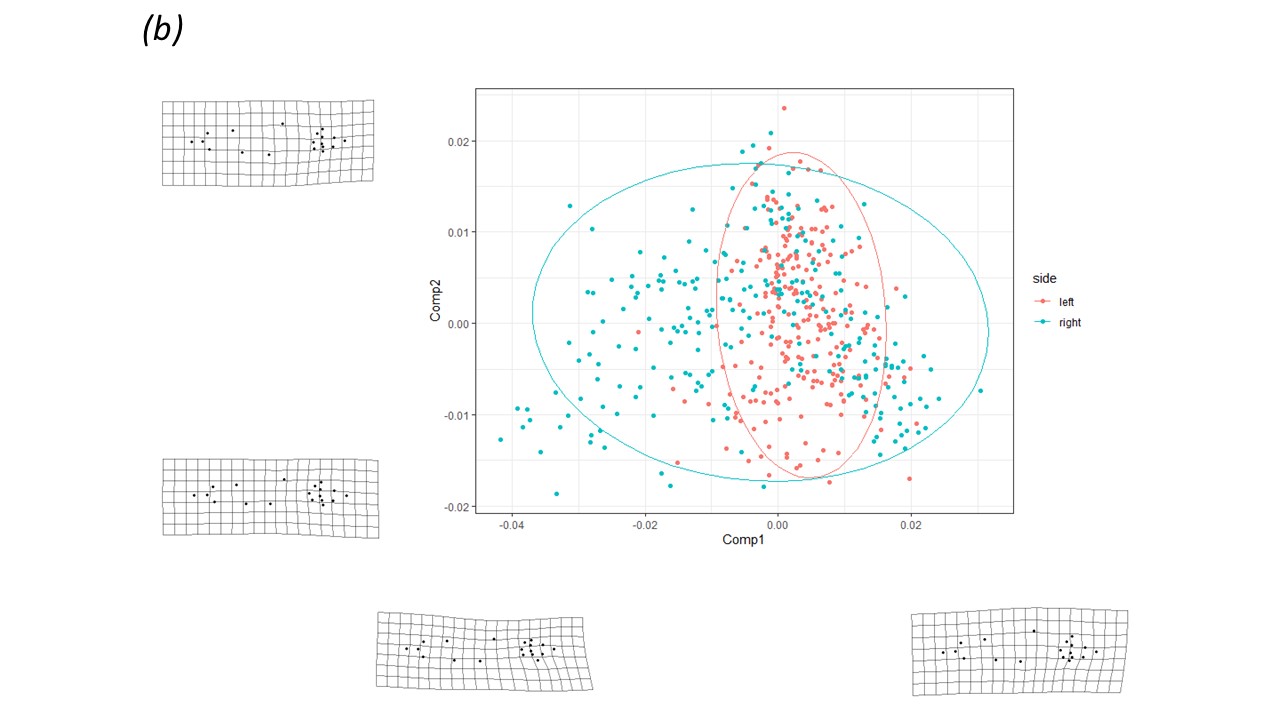

Supplement: Supplementary file 1 — FIGURE S1. Principal component analysis summarising body shape, based on 19 landmarks, of all fish used in this study (a) before and (b) after being adjusted for fish bending. Each point represents a fish, coloured by the side the photograph was taken on, either left (red) or right (blue). Adjustment for fish bending was done using the tpsUtil software. Thin‐plate splines describing the shape summarised by each of the two main principal components at their minimum and maximum are also provided on the x and y axes. As can be seen in the thin‐plate splines, extremes of the principal components in (a) are characterised by bending along the anteroposterior axis, but this effect is removed in (b) after the adjustment. [file JFB-107-1577-s002.zip › Supplementary Figure 1 B.jpg]

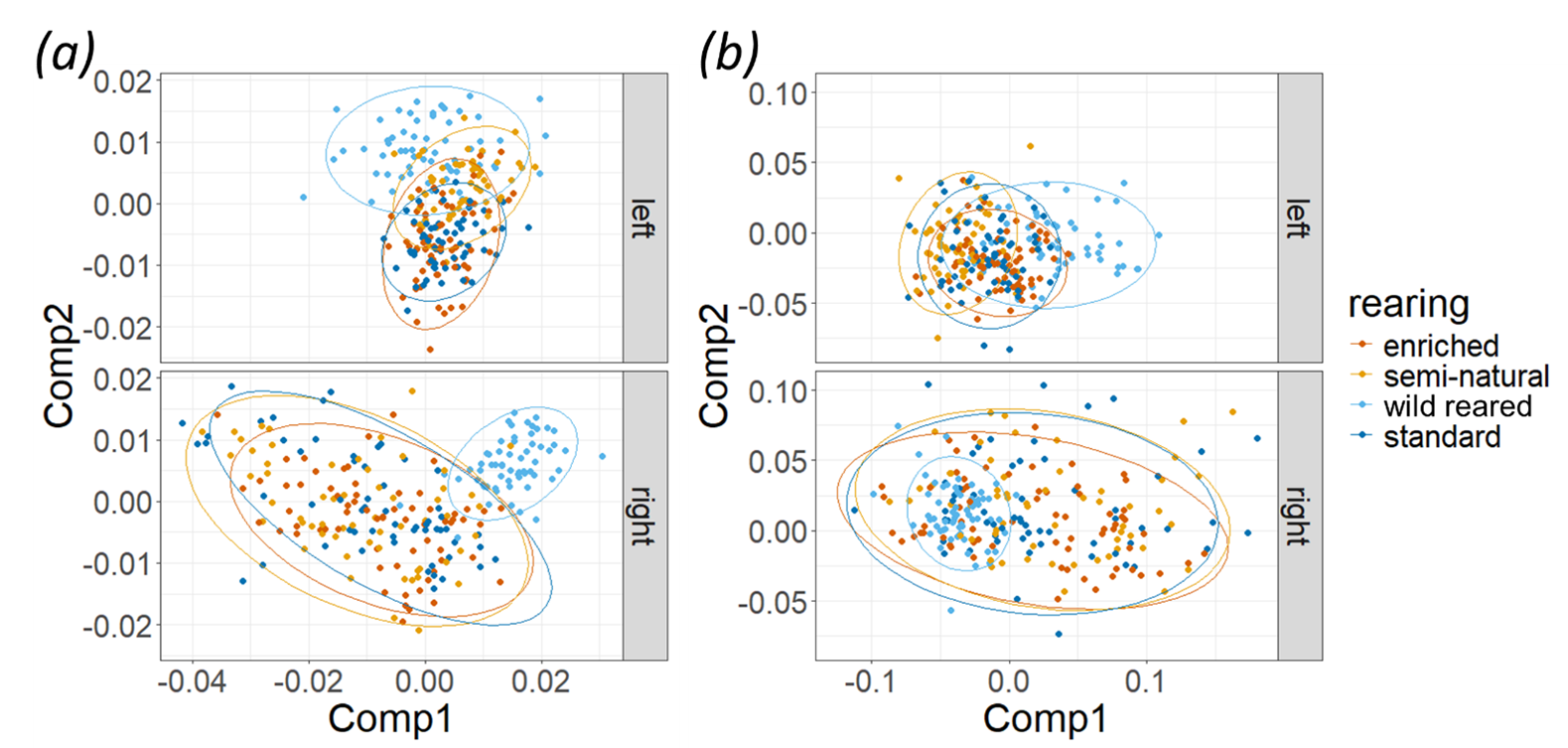

Supplement: Supplementary file 2 — FIGURE S2. Principal component analysis plots from the geometric morphometric analyses assessing (a) body and (b) head shape. Plots are split by the side a photograph was taken and coloured by rearing type. The 95% confidence ellipses can be seen around the data points, coloured by rearing type. These plots look to identify differences in body and head shape caused by rearing alone, regardless of asymmetry. Points closer together indicate a more similar shape. [file JFB-107-1577-s001.png]

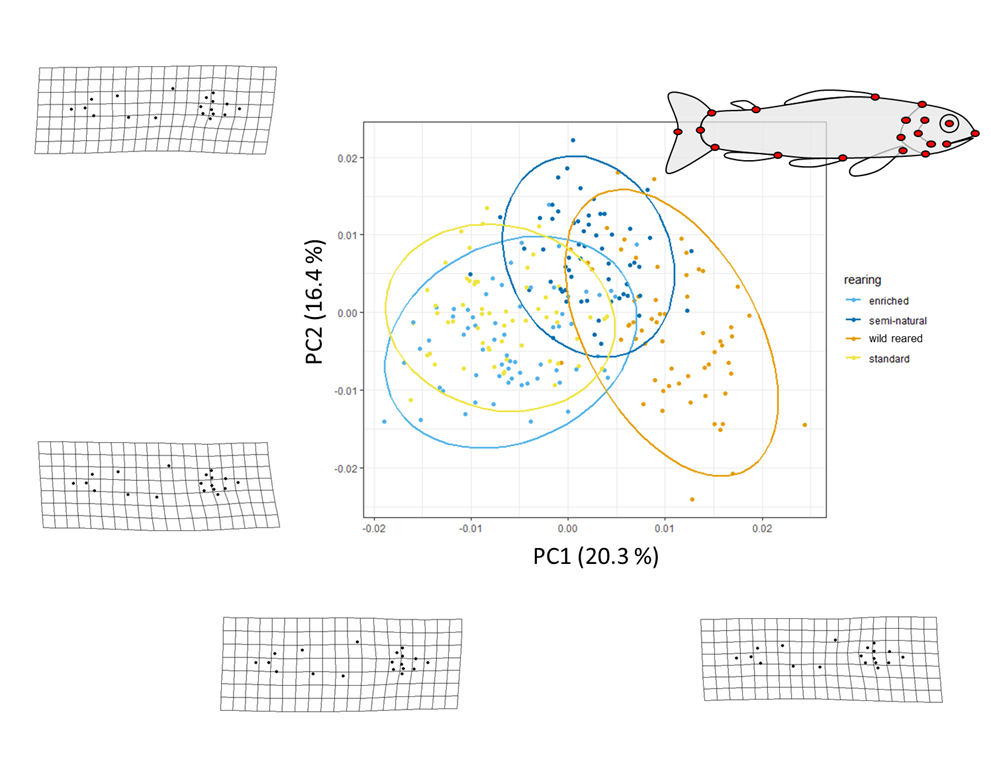

Supplement: Supplementary file 3 — FIGURE S3. Principal component analysis summarising body shape, based on 19 landmarks, of all fish used in this study, but with points only taken from the left side of the fish. The left side was chosen because it displayed less variation in principle component one, as shown in Figure S2. Points are coloured by rearing type. Points closer together indicate a more similar shape. Thin‐plate splines are present along the x and y axes, describing the shape summarised by each of the two main principal components at their minimum and maximum. Finally, a salmon diagram with the position of the landmarks (red points) which formed the basis of the shape analysis is also included. [file JFB-107-1577-s003.png]
